# Senolytics: from pharmacological inhibitors to immunotherapies, a promising future for patients’ treatment

**DOI:** 10.1038/s41514-024-00138-4

**Published:** 2024-02-06

**Authors:** V. Lelarge, R. Capelle, F. Oger, T. Mathieu, B. Le Calvé

**Affiliations:** 1 StarkAge Therapeutics, Campus de l’Institut Pasteur de Lille, 1 rue du Professeur Calmette, 59800 Lille, France; 2 University of Lille, Inserm, CHU Lille, Institut Pasteur de Lille, CNRS, U1283 - UMR 8199 - EGID, 59000 Lille, France; 3 Synlab, 60/62 Rue d’Hauteville, 75010 Paris, France

**Keywords:** Drug discovery, Cell biology

## Abstract

The involvement of cellular senescence in the initiation and propagation of diseases is clearly characterized, making the elimination of senescent cells essential to treat age-related diseases. The development of senolytic drugs demonstrated that targeting these cells limits the deterioration of patients’ condition, by inducing apoptosis. Nevertheless, the first generations of senolytics which has been developed displayed their activities through specific mechanisms and demonstrated several limitations during clinical development. However, the rational to eliminate senescent cells remains evident, with the necessity to develop specific therapies in a context of diseases and tissues. The evolutions in the field of drug discovery open the way to a new generation of senolytic therapies, such as immunological approaches (CAR-T cells, Antibody-Drug Conjugated or vaccines), which require preliminary steps of research to identify markers specifically expressed on senescent cells, demonstrating promising specific effects. Currently, the preclinical development of these strategies appears more challenging to avoid strong side effects, but the expected results are commensurate with patients’ hopes for treatments. In this review, we highlight the fact that the classical senolytic approach based on drug repurposing display limited efficacy and probably reached its limits in term of clinical development. The recent development of more complex therapies and the extension of interest in the domain of senescence in different fields of research allow to extend the possibility to discover powerful therapies. The future of age-related diseases treatment is linked to the development of new approaches based on cell therapy or immunotherapy to offer the best treatment for patients.

## Introduction

Senescence is a natural biological process in which a normal cell blocks its proliferation, changes its morphology, and secretes specific cytokines and chemokines. Several stresses such as oncogene induction, oxidative environment, telomere shortening or radiation exposure are key drivers of senescence induction^1–3^. The accumulation of senescent cells in different organs is a precursor of fibrosis development, neurological disorders, or chronic diseases and the rational to develop specific therapies targeting senescent cells has emerged as a promising therapeutic way^2–4^. Since a decade, senelotic and senomorphic drugs are under evaluation in combination with other treatments in a large panel of diseases. However, the results of clinical trials remain mitigated while the necessity to eliminate senescent cells is still present, highlighting the fact that, several modifications in terms of strategy are essential to improve patients’ lifespan^5^. In this way, new therapeutic strategies had been developed particularly with the evolution of immunotherapies which should provide better results.

In this review, we present the senolytic drugs as a promising way to treat age-related diseases but also their limitations during clinical development. Recently, several new approaches such as specific inhibitors, cell therapies or vaccines have been developed and could extend potential senolytic drugs available on the market in the next few years.

## Targeting Senescent Cell Anti-Apoptotic Pathways (SCAPs)

The development of senolytics to eliminate senescent cells (SnCs) is one of the strategies used to treat age-related diseases. The major problem to eliminate SnCs is their intrinsic resistance to apoptotic stimuli making most cytotoxic drugs inefficient^6,7^. Based on this evidence, the principle of senolytic approaches is to target Senecent Cell Anti-Apoptotic Pathways (SCAPs) by inhibiting or activating proteins which regulate this apoptosis resistance^8,9^. Most of the senolytic drugs have either been identified by repurposing compounds with classical mechanisms of action or have already been used in other fields of research. Among those drugs, the combination of Dasatinib (D), a pan-inhibitor of tyrosine kinase receptors and Quercitin (Q), a flavonoid compound extract from plants targeting BCL-2 family members, is one of the most characterized as well as one of the most used in clinical trials to eliminate senescent cells. Indeed, the combination of D + Q is more effective and allows to target more senescent cell types (compared to the use of each drug alone), by inhibiting the pro-survival pathways induced in senescent cells, notably in idiopathic pulmonary fibrosis (IPF)^10–12^, diabetes and chronic kidney disease^13^ or Alzheimer’s disease^14^. Navitoclax (ABT-263), another inhibitor of the BCL-2 family members, has also been used in many different studies over the years for its ability to eliminate senescent cells by inducing apoptosis. Nevertheless, if its senolytic action has been demonstrated in several diseases such as osteoarthritis^15,16^ as well as in neurodegenerative diseases^17^, the effect of ABT-263 is restricted to senescent cells from specific cell types^18^, leading to the necessity to identify new potential senolytics to target senescent cells in age-related diseases. In this review, we will present different molecules designed to selectively eliminate senescent cells and which are based on new identified targets overexpressed in different senescent models.

### Fisetin

The senolytic activity of Fisetin, a flavonoid polyphenol, has been discovered during an in vitro screen of different polyphenols and its activity has been confirmed in vivo by analyzing the evolution of senescent cells accumulation in progeroid mice^19^. Since this study, several other publications highlighted the potential interest of Fisetin to treat different diseases related to senescence cells accumulation such as kidney fibrosis^20^ or muscular dystrophy^21^. More recently, Fisetin treatment in SARS-CoV-2 infection displayed a significant activity in vivo by eliminating senescent cells and reduced mortality associated with the virus^22^. The safety of the molecule administrated in mice models comparing to other senolytic treatments^23^ push to start more clinical studies in various pathologies (Table 1).

**Table 1:** List of senolytic drugs and their potential use in age-related diseases.

### HSP90 inhibitors

Heat shock protein 90 (HSP90) is a chaperone involved in cellular homeostasis in different physio-pathological conditions, such as cancer. Thus, different inhibitors of HSP90 have been developed and tested in clinical trials to target the protein in different types of cancer. More recently, a study showed that HSP90 is involved in the pathogenesis of pulmonary fibrosis^24^ and more particularly in IPF^25,26^. Indeed, 17-AAG (Tanespimycin) and 17-DMAG (Alvespimycin), two synthetic derivates of Geldanamycin antibiotic, used as HSP90 inhibitors, improve physiological parameters in bleomycin-induced pulmonary fibrosis mice model^25,26^.

More recently, the use of IPI504 (Retaspimycin, a synthetic derivative of Geldanamycin), in an in vitro model of age-related macular degeneration (AMD) on senescent ARPE-19 (human arising retinal pigment epithelial cells), leads to a decrease of SA-β-Galactosidase positive cells, associated with a downregulation of senescence-associated gene expression as well as SASPs secretion, suggesting that in some cases HSP90 inhibitors could also be used as senomorphics^27^.

### P53/MDM2 inhibitors

#### FOXO-DRI

Foxo4 (Forkhead Box O 4) is a transcription factor involved in the insulin signaling pathway and is a regulator of ROS. Baar et al. showed that FOXO4 expression is upregulated in irradiated-induced senescent IMR90 cells and that the protein binds to p53, inhibiting the role of this last one in apoptosis induction. The administration of a synthetic peptide (FOXO4-DRI = FOXO4-D-Retro-Inverso) to avoid the interaction between FOXO4 and p53 leads to a decrease of irradiated-induced senescent fibroblasts viability. Moreover, FOXO4-DRI appears more selective towards senescent cells compared to ABT-737 (an inhibitor of BCL-2 family members). In age-related diseases and more particularly in IPF, mice treated with FOXO4-DRI display less senescent cells leading to a reduction of the progression of fibrosis^28^.

#### UBX0101

This molecule is a p53/MDM2 inhibitor used as a senolytic to treat osteoarthritis (OA). Indeed, UBX0101 treatment on aged mice with post-traumatic OA after surgery reduces the presence of senescent cells, improves articular cartilage, and upregulates the expression of genes involved in new cartilage growth^25^. Moreover, UBX0101 reduces significantly oxidative protein stress and displays pro-regenerative response^29^. Unfortunately, a phase II clinical trial of UBX0101 (NCT03513016) was completed in patients with osteoarthritis and the promising results observed (Table 1), in vitro and in vivo, have not been up to the expectations in treated patients, leading only to a decrease of pain, without a significant improvement of the disease^30^.

#### P22077/P5091

Ubiquitin-Specific Peptidase 7 (USP7) regulates p53 expression by preventing MDM2 degradation from the ubiquitin-proteasome system. The use of P5091 or P22077, two USP7 inhibitors, display a strong senolytic activity in several models of senescence^31^. While the senolytic activity of P22077 has been confirmed in another study^32^, the activity of P5091 is sometimes associated with pro-senescent drug^33^ or senolytic activity.

### Galactose modified prodrugs

#### Duocarmycin

Senescent cells are characterized by high levels of (SA-β-Galactosidase) implying that generation of prodrugs combining a cytotoxic compound linked to a cleavable galactose part should be preferentially processed by those cells. Duocarmycin, a natural antibiotic with cytotoxic activity, has been modified in a Galactose Modified Duocarmycin (GMD) form to target senescent cells. Thus, in the study from Guerrero et al., GMD selectively induces apoptosis in OIS models. Moreover, GMD is also efficient in other types of senescence inductions such as genotoxic stresses, irradiation, and replicative exhaustion. In vivo, GMD eliminates senescent cells and downregulates the expression of senescence-associated genes, in a model of whole-body irradiation^34^.

#### Gemcitabine

Galactose-modified Gemcitabine also called senescence-specific killing compound 1 (SSK1) decreases senescent cells viability in multiple cell types, in mice and human in vitro models as well as in a broad range of inductions such as irradiation, replicative exhaustion, OIS and oxidative or genotoxic stress. Mice treated with SSK1 in a bleomycin-induced pulmonary fibrosis model, harbor less senescent cells within the lungs, associated with a downregulation of senescence-associated genes expression and an improvement of mice physical functions as well as survival rate. Similar results were found in naturally aged mice^35^. Recently, this drug has been used for gastric cancer treatment in combination with a KD4MC inhibitor by applying the “one-two punch” strategy^36^.

### Sodium/potassium pump inhibitors

Senescent cells present a higher concentration of protons to their plasma membrane since this membrane is slightly depolarized compared to non-senescent cells. This characteristic makes cardiac glycosides good candidates as senolytics to target senescent cells, due to their role as specific inhibitors of the NA^+^/K^+^ ATPase pump. Ouabain, a natural compound belonging to the cardiac glycosides’ family, reduces cell survival through apoptotic pathway in human senescent cells induced by genotoxic stress (Etoposide, Doxorubicin and Palbociclib), OIS, or replicative exhaustion. Moreover, digoxin and digitoxin, two other cardiac glycosides usually used in cardiac diseases, decrease IMR90 survival by apoptosis in OIS. In vivo, ouabain-treated mice harbored a decrease of senescent cells in an OIS model, a whole-body irradiation model as well as in aged mice. Moreover, this effect is combined to a downregulation of the expression of senescence-associated cytokines in the second model and an improvement of mice physical condition in the third model, respectively^37^.

### The future of senolytic drug discovery

Most of the senolytic drugs have been discovered by screening libraries of compounds in different models of senescence. Nevertheless, the emergence of Artificial Intelligence (AI) seems to be a promising approach to identify new class of molecules presenting senolytic effects. This method presents several advantages such as to be less consuming in term of time and money as well as its capacity to increase the potential of drugs association, by modeling the combination of different molecules. Recent studies based on machine learning identified different families of compounds already known to target SnCs, such as cardiac glycosides^38^ or molecules mimicking the combination of dasatinib/quercetin^39^, confirming the real potential of this technique. Thus, the use of this approach allowed to identify an original target, the protein kinase DYRK1B, suggesting that an inhibitor of this protein could present a possible senolytic activity^40^. For the moment, the impact of AI on senolytic discovery remains limited but, as in all fields of research, its impact in the near future could be considerable.

**Fig. 1:**
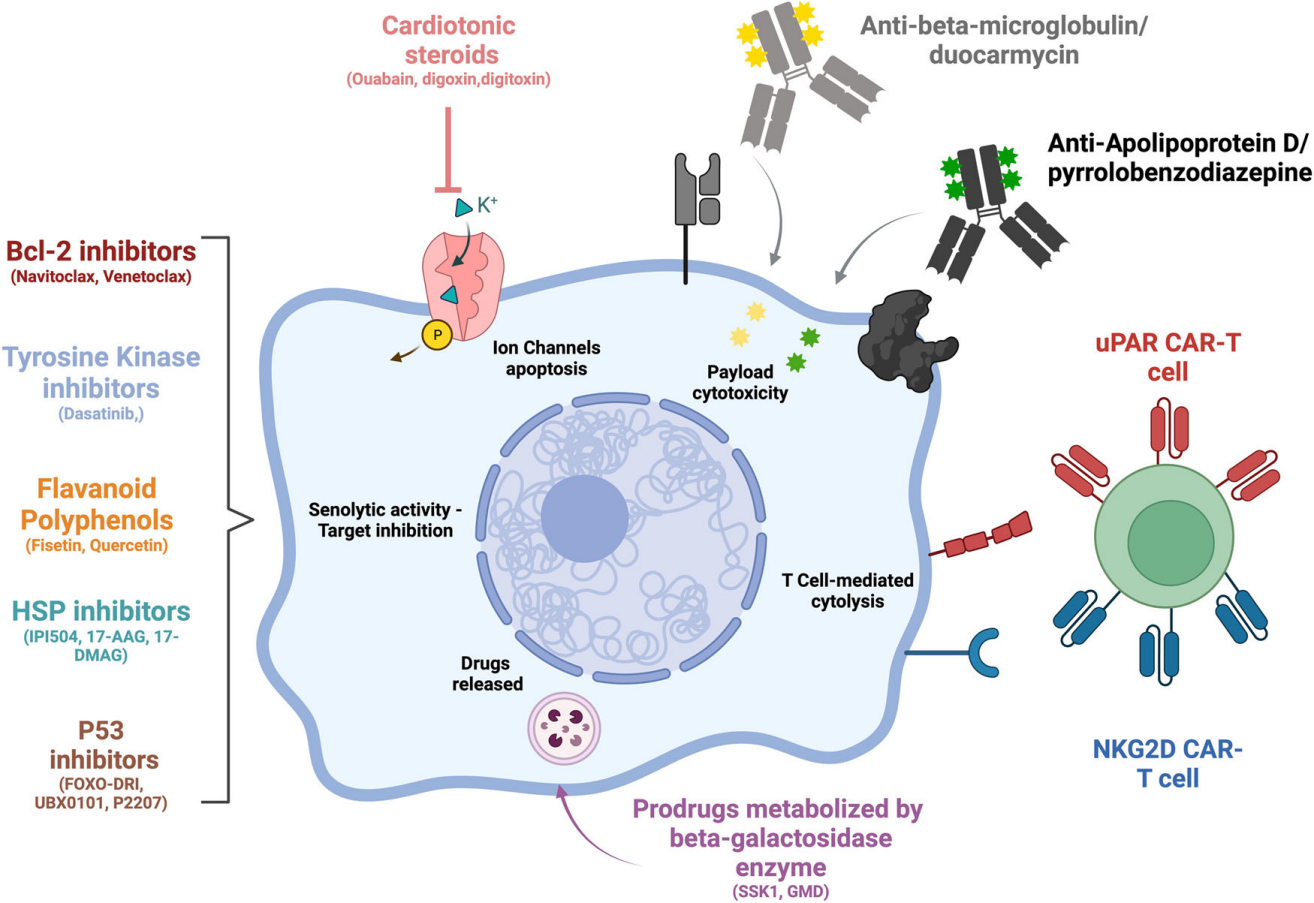
Overview of therapies to eliminate senescent cells. Senolytic approach is based on the expression of specific markers expressed in the cytoplasm or at the plasma membrane of senescent cells. Several therapies have been developed such as pharmacological inhibitors and antibody-drug conjugated or CAR-T cells and display different mechanisms of actions. Created with BioRender.com.

All these drugs are present a demonstrated senolytic acitivity in vitro and in vivo (Fig.1) but their efficiency in human still need to be investigated. To this day, several clinical trials are ongoing in a large panel of age-related diseases, but their validation remains uncertain (Table 1). For most of them (e.g., the cardiac glycosides), the clinical development will be limited due to their toxicity associated with expected side effects in human. The next generation of therapies to eliminate senescent cells should use more selective approaches to avoid detrimental effects on patients. Several new opportunities are under evaluation due to recent promising discoveries in molecular and cell biology. Immunotherapies appear such as the most valuable strategy and could lead to the emergence of a new type of senolytics in a near future.

## Immunotherapies and cell therapies associated with senolytic activity

### Regulation of the immune system

The senescent process directly impacts the cells in which this biological mechanism is activated but also the surrounding cells through the SASP or specific proteins expression at the plasma membrane. Accumulation of SnCs during aging is due to the decrease of immune surveillance, probably related to dysregulation of pathways involved in the immune system activation^41,42^. Different mechanisms have been highlighted and SnCs impact a large panel of immune components such as T-cells, macrophages, or Natural Killers (NK)^43,44^. Immunotherapies belong to the field of research in which the development of specific inhibitors or regulators presents the most significant advances in therapy development. Due to the immunogenic activity of SnCs, it could be interesting to evaluate the senolytic activity of these different drugs.

#### Protein programmed death 1 (PD-1)

PD-1 and its related ligand PD-L1 are one of the major pathways that negatively regulates the activity of immune system. This mechanism has been particularly featured in oncology through the downregulation of immune protection during tumor progression. The development of antibodies, against PD-1 or PD-L1 represents one of the greatest advances to date in the treatment of cancer. In the context of senescence, PD-1 and PD-L1 are overexpressed in several in vitro models induced by drugs or related to normal aging phenotype^45^. This overexpression is also significant in different organs (lung, kidney, or liver) in aged mice and the senescent phenotype is reversed by treating mice with an anti-PD-1. The impact of PD-1 expression is also related to the aging of immune cells in the development of diabetes^46^. In this study, an extensive mapping of peripheral blood cells of patients which suffered from type 1 diabetes revealed the expression of parameters related to aging and associated with PD-1 expression in memory T-cells. In this case, an anti-PD-1 could be considered for the treatment of type 1 diabetes. The rational to target PD-1/PD-L1 axis in senescence is evident and the large panel of drugs approved to target this pathway will probably accelerate the development of this strategy.

#### CD47

the surface protein CD47 is a receptor that binds to signal-regulatory protein alpha (SIRPα) or thrombospondin-1 (TSP-1) and acts as a signal to inactivate the phagocytosis in macrophages. The downregulation of macrophages activity through the overexpression of CD47 is already known as a mechanism for cancer cells to escape the immune system. In the context of senescence, SnCs seem to use the same mechanism to avoid their elimination by macrophages^47^. In vitro and in vivo, CD47 is overexpressed in senescent models and blocks their recognition by macrophages. The inhibition of SIRPα- CD47-SHP-1 axis by using CD47 antagonist, under clinical evaluation, leads to SnCs elimination and maintain the homeostasis of tissues. This type of therapy is widely used in cancer research and could be investigated as a senolytic approach for chronic diseases. Recently, a mimetic peptide of TSP-1 called 4N1Ks has been developed as a senolytic therapy and confirm the high interest to investigate CD47 pathway in senescence^48^.

**Fig. 2:**
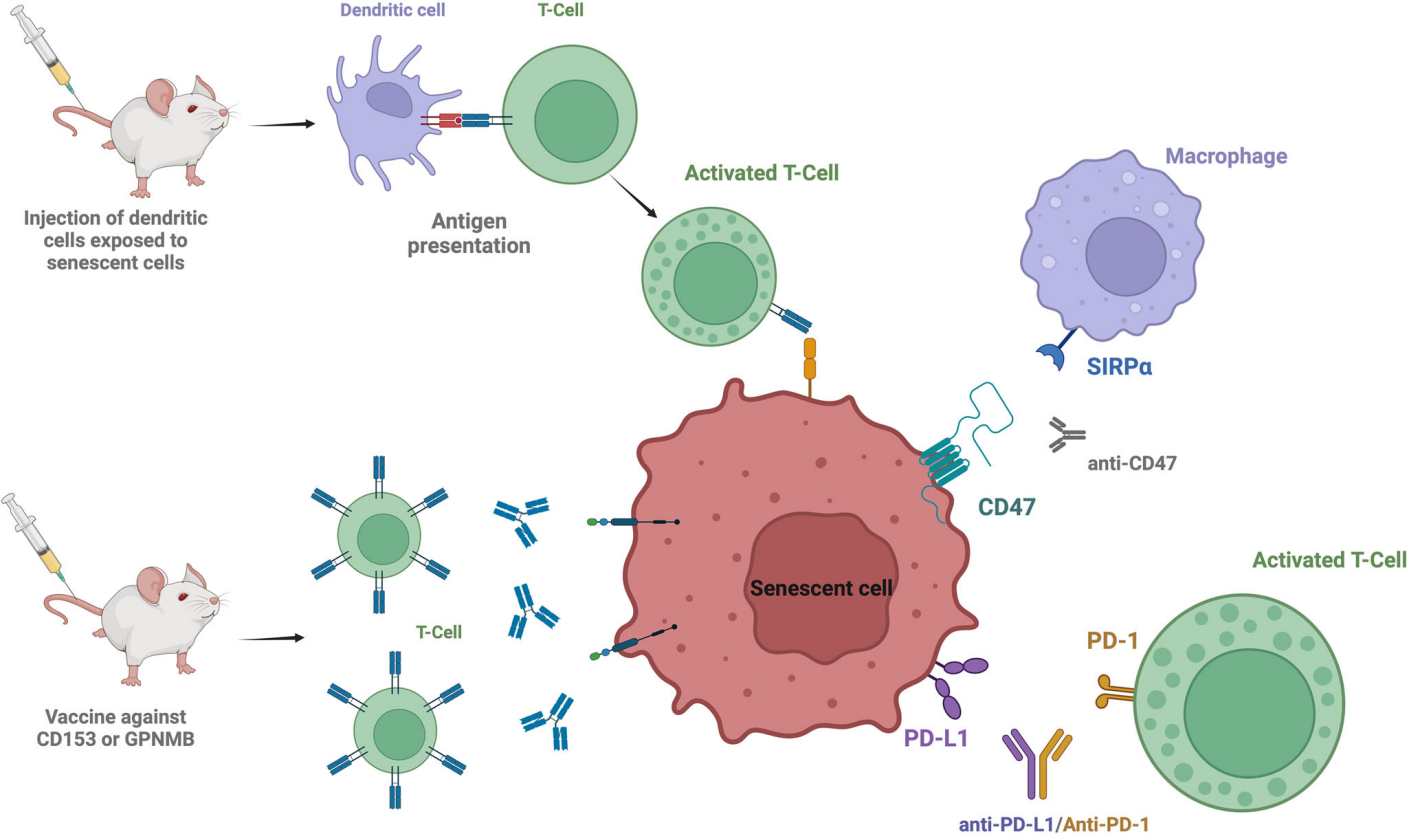
Vaccine and immunological approaches used to target specifically senescent cells. Activation or modulation of the immune system trigger the recognition and the elimination of senescent cells. Created with BioRender.com.

This field of research is probably the most promising because: 1/ immunology has undergone incredible development in the last ten years and 2/ the number of treatments which modulate the immune system efficiency is massive. Different pathways have been recently identified to play a key role in the escape of the immune system in senescence, highlighting the potential development of significant senolytic therapies in the future (Fig. 2).

### Antibody-drug conjugates (ADCs)

#### Beta-microglobulin (B2M)

To improve the efficiency of senolytics, a new approach is urgently needed to eliminate senescent cells and fight age-related diseases. In a recent proof-of-concept, Poblocka et al. explored the feasibility of ADCs as targeted therapies associated with senolytics. They analyzed different senescent tissues (brain and skin) and identified B2M as a potential surface marker of senescent cells, associated with p16 expression. Based on these observations, an ADC conjugated to duocarmycin through a cleavable enzymatic linker was designed against the B2M protein and used as a senolytic therapy. Senescence induction in colon (HTC116) and bladder (EJ) cancer cell lines, followed by an incubation with the B2M-ADC, led to the clearance of 35% of senescent cells. The cell death induced by the B2M-ADC does not seem to be led by apoptosis but rather by necrosis^49^.

#### Apolipoprotein D

Another study highlighted the potential of ADC as a senolytic strategy by combining an antibody against Apolipoprotein D (ApoD) and an inhibitor of DNA synthesis called pyrrolobenzodiazepine (PBD)^50^. This molecule is an antibiotic and presents an anticancer activity by inhibiting DNA synthesis. Despite a promising in vitro antitumor activity, the development of this therapy faced off serious side effects, limiting its potential development as anticancer therapy. With the development of ADC approach, PBD has been used as a payload associated with specific antibodies in the treatment of different diseases, limiting the high toxicity associated with this molecule. During senescence, ApoD is overexpressed in several age-related diseases, notably in neurodegenerative disorders, fibrosis, or cardiovascular disease. Skin senescence displays a higher level of ApoD expression compared to young tissues, making this protein as a specific marker of aging. The combination of PBD and an antibody against ApoD demonstrated a senolytic readout by eliminating senescent fibroblasts without any effect on young cells. By combining ApoD antibody and PBD as an ADC therapy, Takaya et al. selectively eliminated senescent skin fibroblasts in vitro and in vivo in a model of aged mice and increased the mechanical properties of skin in aged population. This method repurposes PDB as a potential senolytic therapy in age-related disorders but also in cancer senescent cells elimination. This therapy obviously needs to be more investigated to avoid side effects in normal tissues but the combination of specific senescence markers to cytotoxic drugs is a potential promising therapy.

The ADC approach could offer a second life to drugs presenting a high cytotoxicity or a lack of selectivity (Fig. 1). In this way, ADCs have a promising potential as targeted senolytic drugs to fight against age-related diseases. Moreover, the association of specific markers of SnCs and senolytic drugs could be a great combination in the treatment of age-related diseases.

### CAR-T cells therapy

#### Urokinase-type plasminogen activator receptor (uPAR)

Different senolytics have been developed in the antibody or small drug fields but nothing regarding senescent cells due to the lack of identification of specific surface targets on these cells^51^. However, this view has been recently challenged with the identification of an uncommon biomarker between senescence and healthy cells named urokinase-type plasminogen activator receptor (uPAR) by Amor et al. ^52^. In this article, the authors showed that uPAR is expressed in senescence cells, both in in vivo and in vitro models (liver fibrosis, pancreatic intraepithelial neoplasia, senescent melanocytes, or lung cancer) correlated to a lack of expression in healthy tissue, except for the bronchial epithelium and a subset of immune cells in which a weak expression of uPAR was detected. The efficacy of uPAR-targeted CAR-T cells to remove senescent cells in vivo was investigated in different mice models with different age-related diseases, including cancer and liver fibrosis, and showed a significative efficiency. In an OIS model induced by Nras^G12v^ overexpression in hepatocytes of immunodeficient Nod Scid Gamma (NSG) mice, senescent cells were successfully eliminated when treated with the anti-uPAR CAR-T cells. A second publication confirmed the efficiency of this CAR-T therapy notably by improving glucose metabolism and physical ability of treated mice. The most interesting point of this study is the preventive effect of this approach due to a persistence of efficiency during the time after a single injection of the treatment^53^.

#### NKG2D

The regulation of the immune system activity by senescent cells is a key factor by which age-related diseases are developing in the human body. One of the mechanisms by which senescent cells escape from the immune response is made through the overexpression of the histocompatibility antigen alpha chain E (HLA-E) that bind to NKG2A, an inhibitor receptor expressed on Natural Killer cells (NK)^54^ and CD8^+^ T cells, leading to the inhibition of SnCs recognition. Nevertheless, the expression of these NKG2D ligands open the way to use CAR-T cell therapy based on the recognition of these molecules overexpressed on the cell surface of senescent cells. Yang et al. ^55^ used a classical NKG2D CAR-T in different models of senescence to illustrate the specificity of this approach as senolytic approach. In vitro, NKG2D CAR-T cells selectively eliminate senescent fibroblasts induced by different stresses as well as in vivo, in mice and non-human primate aged models where the effects of this cell therapy are significant without any toxicity observed.

CAR-T approach is one of the most promising therapies in cancer research but the possibility to extend to senescence field is challenging and could provide a long-term protection against age-related diseases. But there are several obstacles to the development of this senolytic approach such as the long-term effect of CAR-T cells therapy with possible aspecific toxicity in different organs^56^, the cost and the difficulty during process development^57^ or the selection of non-exhausted T cells to generate efficient therapy^58,59^.

### 3- Senolytic vaccines

Vaccine-based strategies have been also examined for the development of senolytics to treat age-associated disorders. Contrarily to the other methods listed before, vaccine-based approaches do not only aim to target senescent cells in tissues, but also senescent immune cells, which can be responsible for age-related diseases^60^. Beside these senolytic vaccines, other senotherapeutic vaccines intent either to boost the release of anti-aging molecules, or to neutralize other molecules identified as pro-aging factors. Other methods of vaccination based on cellular vaccines have also been proposed for senotherapeutical uses (Fig. 2). In the following part, we will describe the different vaccine-based strategies of senotherapeutics that have been studied, and we will show the limitations of some of them to be used into clinics.

#### The Transmembrane Glycoprotein NMB (GPNMB) vaccine

The expression of this transmembrane protein was found to be increased in aging (e.g., human mesenchymal stem cells from old donors) and in some age-related pathologies, such as osteoporosis^61^. GPNMB expression was also increased in senescent human umbilical vein endothelial cells, in endothelial cells from old mice, and in several other tissues such as bone marrow and lungs^62^, suggesting that GPNMB could be a potential target in senescence-related pathological conditions. Indeed, in mice fed with a high fat diet (HFD) and vaccinated against GPNMB, Suda et al. observed a decrease in senescence biomarkers expression and an increase of glucose tolerance. Cellular analysis of the adipose tissue from HFD mice showed that GPNMB^high^ cells, primarily associated with senescent endothelial cells, are significantly reduced due to the effect of the vaccination. Interestingly, only a small part of cells expressing low level of GPNMB, were eliminated by the vaccinated mice^62^. Moreover, this treatment showed also promising results when administered to a mouse model of atherosclerosis (ApoE KO mice). Eight weeks post-vaccination, a decrease in number and size of atherosclerotic plaques was reported, along with a decrease of GPNMB senescent cells number. In addition, the vaccine improved the physical activity of middle-aged mice (50 weeks old) and extended the median lifespan of ZMPSTE24 KO mouse (model of progeria), an extreme model of accelerated aging^62^. Together these findings suggest that the elimination of senescent cells through vaccination targeting GPNMB-expressing cells could be beneficial to health span and possibly extend lifespan.

Even though developing a vaccine against GPNMB positive cells seems to be beneficial, the challenge remains to only deplete cells with the highest expression of the protein but not the cells expressing it at a physiological level^63^. Moreover, GPNMB has protective roles in the body by contributing to the resolution of inflammation and by inhibiting T cells activation^64,65^. Therefore, the development of this vaccine in clinical trials could be more complicated than in in vivo experiment, given the diverse physiological functions of GPNMB in normal cells.

#### The CD153 vaccine

Immunosenescence, defined as the decreased capacity of the immune system to respond effectively to infections or vaccines in the elderly, is caused by the senescence of immune cells^66^. For example, senescent T-cells are accumulated in visceral adipose tissues in obesity^67^ and produce proinflammatory cytokines, leading to chronic inflammation, metabolic disorders, and cardiovascular diseases^68,69^. Therefore, elimination of senescent immune cells, including those accumulating in tissues, has been proposed to improve the immune system activities. As such, a vaccine targeting and destroying selectively CD153-expressing senescent T-cells is promising since it improves the glucose metabolism in obese mice^70^. The vaccine did not only reduce the number of senescent T-cells, but also prevented the accumulation of adipose senescent T-cells. In HFD mice, the vaccine was able to prevent glucose intolerance. This approach of senescent T-cells elimination has a great potential to restore immune system function and therefore, to protect the aged animal against numerous aging-associated disorders^70^. These findings highlight the efficiency of senotherapeutic vaccines targeting senescent immune cells.

#### Senescent cancer cells as vaccine therapy

Therapy-induced senescence is associated with anticancer treatment and promotes deleterious effects such as relapse, drug resistance or immunosuppression^71^. But, on the other hand, the induction of senescence during cancer treatment could be used as a potential mechanism to induce immune reaction against cancer cells. Recently, Liu et al. ^72^ highlighted the fact that senescent cancer cells could be used as cancer vaccine by activating the antitumor immunity. Indeed, they showed that dendritic cells are activated in vitro in co-culture with senescent cancer cells. More interestingly, injection of senescent cancer cells or dendritic cells activated by senescent cells in mice protects from tumor engraftment, induces significant tumor regression, and potentiates immunotherapies. In the same way, the purification of senescent cancer cell-derived nanovesicles (SCCNVs) could be used as vaccine strategy for cancer treatment^73^. Indeed, the SCCNVs could be produced by inducing senescence ex vivo in the cancer cells of patients and after several steps of purification, SCCNVs could be re-injected into the patients. This process avoids adding external molecules since interferon gamma (IFN-γ) and tumor necrosis factor-alpha (TNF-α), two cytokines associated to the SASP, act as natural adjuvant by promoting an immunoreaction. The SCCNVs present a large panel of specific antigens to improve vaccine immunogenicity and activate the CD8^+^ population. Generating and injecting SCCNVs from B16F10 senescent cancer cells, induced by doxorubicin treatment, alter significantly by slowing down tumor progression but also in a lung metastasis model by limiting tumor development. This approach needs to be validated in terms of potential side effects associated with the induction of the immune reaction and more particularly with the non-specific antigens present at the surface of SCCNVs. In another study, Marin et al. ^74^ showed that the immunization with senescent cells is not only possible with senescent cancer cells but also with normal senescent cells. The phenotype of senescent cells is associated with the presentation of specific peptides implicated in the activation of CD8^+^ lymphocytes. The immunopeptidome of the senescent phenotype is associated with 70 peptides which makes senescent cells immunogenic and potentially targetable by immune cells. Moreover, the authors extended this mechanism to senescent cancer cells and demonstrated that these cells are more efficient than immunogenic cell death to induce immune system protection against tumor relapse. This strategy could be used as an efficient strategy of vaccination against cancer development.

## D- Perspectives

The elimination of senescent cells in a pathological context appears more complicated than anticipated, especially because of the physiological function of senescence in normal tissues. Moreover, the senescence phenotype depends on specific pathologies or tissues, making the identification of selective markers essential for the development of new senolytic drug families. Since few years, targeted therapies emerged as a promising senolytic approach, and several molecules are under clinical evaluation. These therapies display higher efficiency compared to classical senolytic drugs (BCL-2 or tyrosine kinase inhibitors) but for some of them, such as NA^+^/K^+^ ATPase inhibitors, clinical development could be complex in the light of strong cardiotoxicity associated with this class of molecules. More recently, scientific advances in the field of antibody or cell therapy allowed the development of a new generation of senolytic strategies. For example, ADC combining antibody and cytotoxic drugs could be a promising strategy to specifically target senescent cells with non-specific compounds. In the same way, CAR-T cells therapy targeting uPAR protein displays a huge in vitro and in vivo senolityc activity and could be considered in a close future as an alternative solution to definitively eradicate senescent cells in rejuvenation or to treat age-related diseases. Like ADC, CAR-T cells therapy needs to be very specific because of the risk of off-target that could lead to strong side effects even though expected results should be potentially higher regarding its long-term efficacy. The development of bispecific antibodies or dual CAR-T approaches could probably be a key factor for valuable therapies. Finally, vaccines represent the most recent type of treatment used to eliminate senescent cells. Several strategies have been explored through the classical development of vaccines against targets or directly by exposing immune cells to different senescent phenotypes. The associated preclinical studies highlighted promising activities to fight against age-related diseases but also in cancer treatment. In addition, the development of immunopeptidome analysis should provide successful targets and open the way to a new generation of senolytic treatments.

The necessity to eradicate senescent cells in pathological contexts remains a high priority to develop innovative therapies in a large scale of diseases. In the past twenty years, and mainly by repurposing drugs use, molecules have been identified but to date, clinical trials display moderated efficiency. After a first line of senolytic drugs tests, several new treatments targeting specific proteins expressed selectively during senescence or based on new technical developments will pave the way of preclinical research. However, development of CAR-T cells or vaccine therapy as senolityc treatment remain, for the moment, difficult because the permanent activation of these therapies could lead to several side effects and potentially eradicate “positive” activity of senescence.
